# Glucose-Dependent Insulinotropic Polypeptide Receptors Are Expressed in the Lateral Septum and Reduce Electrically-Evoked Dopamine Release as well as the Ability of Cocaine to Increase Extracellular Dopamine

**DOI:** 10.1021/acschemneuro.5c00954

**Published:** 2026-06-01

**Authors:** Anna Marie Buchanan, Sonja Virkus, N. Dalton Fitzgerald, Cesar E. Hernandez, Kirk M. Habegger, Jeremy Day, Troy A. Hackett, J. Andrew Hardaway, Aurelio Galli

**Affiliations:** † Department of Surgery, 9968 University of Alabama at Birmingham, Birmingham, Alabama 35294, United States; ‡ Department of Psychiatry & Behavioral Neurobiology, University of Alabama at Birmingham, Birmingham, Alabama 35294, United States; § Department of Neurobiology, University of Alabama at Birmingham, Birmingham, Alabama 35294, United States; ∥ Department of Medicine, University of Alabama at Birmingham, Birmingham, Alabama 35294, United States; ⊥ Department of Hearing and Speech, 12328 Vanderbilt University Medical Center, Nashville, Tennessee 37232, United States

**Keywords:** GIPR, GLP-1R, cocaine, dopamine, lateral septum, D2R

## Abstract

Tirzepatide, a dual agonist of the glucagon-like peptide 1 receptor (GLP-1R) and glucose-dependent insulinotropic polypeptide receptor (GIPR), represents a new class of medication for obesity and type II diabetes treatment. Using laboratory mice, we show that GIPRs are present in the lateral septum (LS) in cells also expressing GLP-1R, particularly in the dorsolateral LS. Likewise, we show the coexpression of the dopamine (DA) D2 receptor (DRD2) in LS cells with GLP-1R in both the dorsolateral and intermediate portions of the LS. Using fast-scan cyclic voltammetry, we demonstrate that systemic GLP-1R or GIPR agonist treatment reduces both electrically evoked DA release in the LS and the ability of cocaine to increase extracellular DA levels. These data unveil a new central role for GIPR signaling and identify a potentially important cell type expressing both GLP-1R and GIPR upon which tirzepatide or other dual agonists may modulate DA homeostasis in the brain.

## Graphical abstract

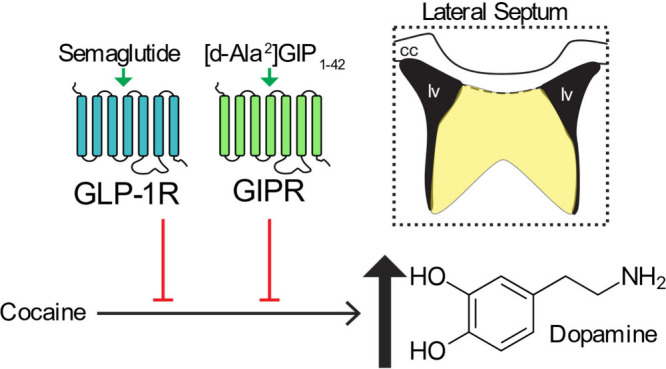

## Introduction

Cocaine is a psychostimulant that in 2022 was involved in 29.7% of overdose deaths within 30 states that report data to the Centers for Disease Control and Prevention (https://www.cdc.gov/overdose-prevention/data-research/facts-stats/sudors-dashboard-fatal-overdose-data.html). To date, there is no FDA-approved pharmacotherapy for cocaine use disorder (CUD) because safe targets regulating addictive behaviors are limited and we lack a comprehensive understanding of their molecular mechanisms. Glucagon-like peptide 1 (GLP-1) and glucose-dependent insulinotropic polypeptide (GIP) are incretin hormones that act through both peripheral and central mechanisms to regulate glucose/energy homeostasis and feeding behavior.^1^ Pharmacological activation of GLP-1 receptors (GLP-1Rs) and GIP receptors (GIPR) decreases hedonic food intake and preference.^1^ Excitingly, systemic GLP-1R agonists attenuate amphetamine- and cocaine-induced locomotion in rodent models.^2,3^ Furthermore, pretreatment with GLP-1R agonists diminishes the rewarding effects of amphetamine and cocaine in a conditioned place preference (CPP) test.^2,4^ Previously our lab demonstrated that a systemic GLP-1R agonist attenuates both the rewarding properties of cocaine, as well as cocaine-mediated increases in extracellular dopamine (DA) in the lateral septum (LS).^5,6^ The LS is a brain region with high expression of GLP-1R.^7^ Interestingly, within the LS moderate GIP immunoreactivity has been previously observed.^8^ The LS is comprised mainly of GABAergic neurons^9^ that projects to different brain regions, including the ventral tegmental area (VTA), a DA node necessary for the formation of cocaine conditioned place preference (CPP)^10^ and cocaine seeking.^9^ The LS has been associated with reward processes^11^ as it relays information to the VTA.^9^ Recent pharmacological advancements aimed at reducing obesity and hedonic feeding have been achieved by the single molecule dual GLP-1R/GIPR agonist, tirzepatide (Mounjaro, Zepbound), that curbs appetite and hedonic eating with minimal side effects.^12^ These findings have inspired our hypothesis that GIPRs are expressed on neurons in the LS and in a subset of neurons that also express GLP-1Rs. They also raise the possibility that combined agonism of GLP-1Rs and GIPRs plays a fundamental role in the hedonic response to drugs of abuse (e.g., cocaine).

## Results and Discussion

Here, we show that *Gipr* expression is enriched in both the dorsal lateral septum (LSd) and intermediate lateral septum (LSi), brain regions that coexpress *Glp1r* (Figure 1A–G). Interestingly, in the LSd, 37.1% of *Glp1r* positive neurons coexpress *Gipr* (Figure 1D,H), whereas in the LSi, *Gipr* is expressed in 18.1% of *Glp1r* positive neurons (Figure 1G,I). Considering that GLP-1Rs are expressed throughout the brain, and many GLP-1R-expressing nuclei have been implicated in regulating the motivational and hedonic properties of food,^13^ the enrichment of *Gipr* in *Glp1r* positive neurons suggests a potentially fundamental role of GIPR in regulating neurocircuits associated with reward processes. We also found that a portion of *Glp1r* positive neurons coexpress DA D2 receptors (*Drd2*) mRNA in both the LSd (Figure 1L,M,Q) and LSi (Figure 1O,P,R). These data suggest a feasible interplay between GLP-1R and GIPR regulation of GABAergic function and synaptic DA release from afferent fibers in the LS (e.g., from the VTA).

**Figure 1:**
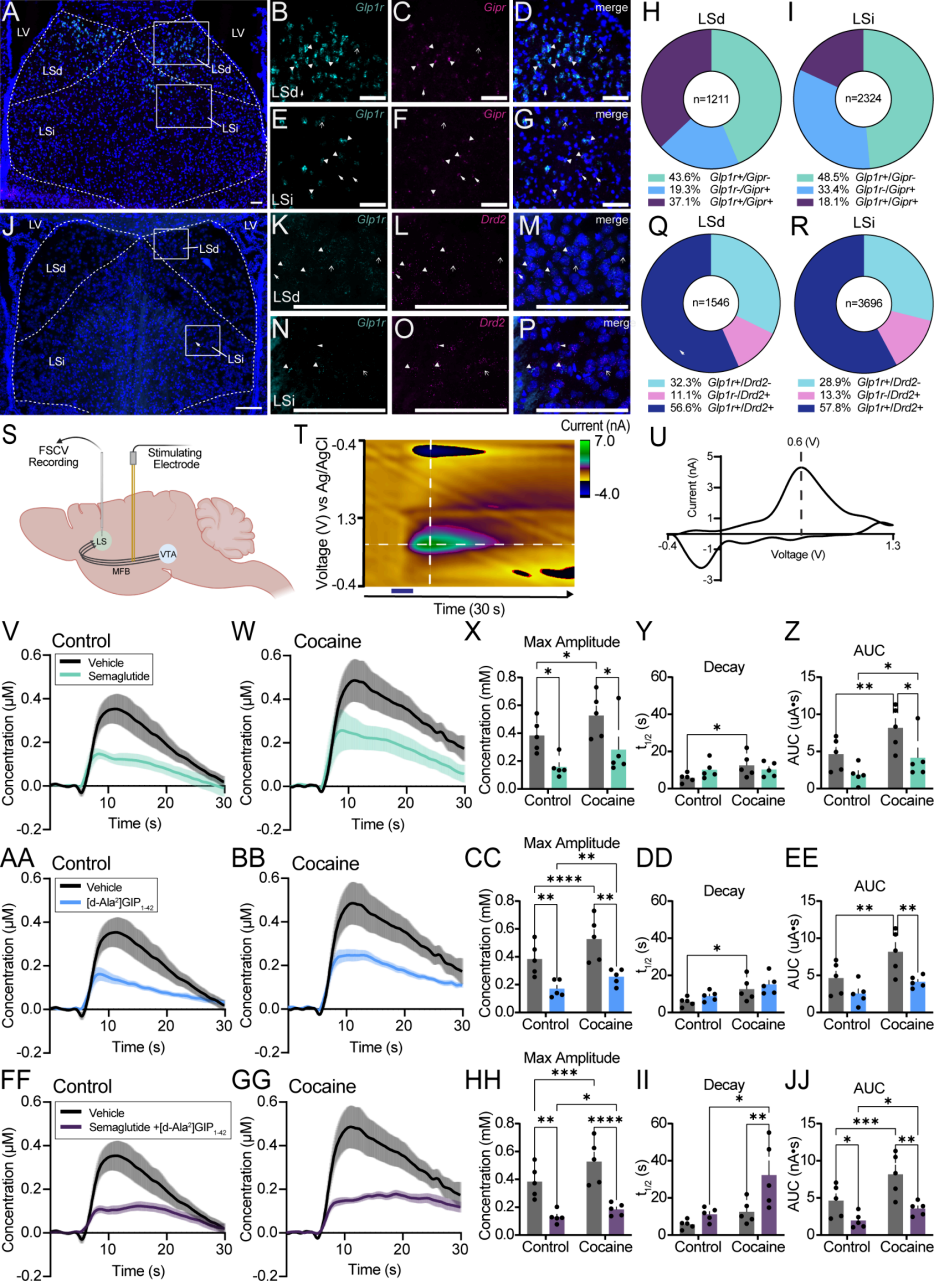
**GIPR and GLP-1R agonism inhibits electrically evoked DA release in LS and the ability of cocaine to increase extracellular DA.** (**A**) Field view of LS with *Glp1r* in cyan and *Gipr* in magenta. Top square: enlarged, single channel view for the LSd shown in (**B,C**) and merged in (**D**); bottom square: enlarged, single channel view for the LSi shown in (**E,F**) and merged in (**G**) (scale bars A–G = 100 μm). Quantification of percentages of *Glp1r*, *Gipr* and doubly labeled cells in the LSd (**H**) and LSi (**I**). (**J**) Field view of LS with *Glp1r* in cyan and *Drd2* in magenta. Top square: enlarged, single channel view for the LSd shown in (**K,L**) and merged in (**M**); bottom square: enlarged, single channel view for the LSi shown in (**N,O**) and merged in (**P**) (scale bars J–P = 200 μm). Quantification of percentages of *Glp1r*, *Drd2* and doubly labeled cells in the LSd (**Q**) and LSi (**R**). (**S**) Schematic for FSCV. (**T**) Colorplot for DA release in the LS. (**U**) CV. Average DA concentration curves of mice receiving injections of vehicle and semaglutide before (**V**) and after (**W**) cocaine. Quantification of max DA release (**X**), *t*_1/2_ of the DA reuptake (**Y**), and AUC (**Z**) for saline and semaglutide administration before and after cocaine. Average DA concentration curves of mice receiving injections of vehicle and [d-Ala^2^]­GIP_1–42_ before (**AA**) and after (**BB**) cocaine. Quantification of max DA release (**CC**), *t*_1/2_ of the DA reuptake (**DD**), and AUC (**EE**) for saline and [d-Ala^2^]­GIP_1–42_ administration before and after cocaine. Average DA concentration curves of mice receiving injections of vehicle and the combined dose of semaglutide and [d-Ala^2^]­GIP_1–42_ before (**FF**) and after (**GG**) cocaine. Quantification of max amplitude of DA release (**HH**), *t*_1/2_ of the DA reuptake (**II**), and AUC (**EE**) for saline and combined agonists before and after cocaine. Statistical tests are listed after the Methods section.

To determine whether GLP-1/GIP signaling regulates DA release in the LS, we adopted fast scan cyclic voltammetry (FSCV) (Figure 1S) which enables the electrochemical detection of DA at a high temporal resolution. We elicited DA release in the LS by stimulation of the medial forebrain bundle (MFB) (Figure 1S). Figure 1T shows a 3D colorplot of stimulated DA release where the vertical line denotes the applied potentials used to generate a cyclic voltammogram (CV) (Figure 1U) to identify DA. Mice received s.c. injections of either vehicle (Control, gray), semaglutide (a GLP-1R agonist; green), [d-Ala]­GIP_1–42_ (a GIPR agonist; blue), or a combined dose of semaglutide and [d-Ala]­GIP_1–42_ (purple) 5 h. prior to cocaine i.p. administration (Figure 1V-Z,AA-EE,FF-JJ, respectively). All three treatments significantly decreased the maximum electrically evoked DA release with respect to vehicle (Figure 1X,CC,HH). In the absence of cocaine, neither single or combined semaglutide and [d-Ala]­GIP_1–42_ treatment had an effect on DA clearance (Figure 1Y,DD,II). Furthermore, among treatments, only semaglutide plus [d-Ala^2^]­GIP_1–42_ significantly reduced the amount of DA released (AUC) in the absence of cocaine (Figure 1Z,EE,JJ); Interestingly, all three treatments significantly reduced the ability of cocaine (10 mg/kg) to enhance maximal electrically evoked DA release (Figure 1X,CC,HH) and the overall amount of extracellular DA as quantified by area-under-the-curve (Figure 1 Z,EE,JJ). However, neither semaglutide nor [d-Ala^2^]­GIP_1–42_ alone impacted DA clearance in the presence of cocaine (Figure 1Y,DD). Instead, combined semaglutide and­[d-Ala^2^]­GIP_1–42_ treatment augmented the ability of cocaine to inhibit DA clearance (Figure 1II). These data demonstrate the ability of both GLP-1R and GIPR systemic agonism to modulate extracellular DA in the LS under basal conditions and in the presence of cocaine.

One limitation of these studies is that our sample size might not have been sufficient to detect potential regulation of DA clearance by GIPR and GLP-1R agonism. Future studies will address this limitation and test alternative mechanisms (GLP-1R/GIPR actions in other brain nuclei). In addition, we did not hypothesize sex differences in GIPR/GLP-1R/DRD2 expression and DA function specifically in the LS, so these experiments were mainly performed in male mice. It will be important to address potential sex difference in future studies.

Understanding the role of incretin hormones in the limbic system is essential for treating diseases associated with disrupted hedonic responses. We show that GIPRs are expressed in the LS and colocalize, at least in part, with cells expressing GLP-1Rs. Systemic GIPR agonism significantly inhibits maximum electrically evoked DA release as measured by FSCV in anesthetized male mice. Notably, we show that combined GLP-1R and GIPR agonism robustly attenuates the ability of cocaine to augment the amplitude of the DA signal produced by electrical stimulation. Considering that psychostimulant exposure causes long-lasting increases in extracellular DA in the LS,^11^ and that LS function is required for the expression of cocaine CPP^10^ and cocaine seeking,^9^ our data point to combined GLP-1R/GIPR agonism as a potential treatment for CUD. Furthermore, we show that systemic GIPR agonism alone is sufficient to regulate LS DA dynamics upon cocaine exposure. Future studies should explore the role of local septal activation of GIPR signaling in animal models of CUD in both female and male mice. In this study, we also define a novel signaling pathway (i.e., GIPR signaling) that inhibits DA release in the LS. It is tempting to speculate that these data point to a new mechanism of how tirzepatide might regulate caloric intake and food preference.

## Methods

### Animals and Surgery

All animal procedures were approved by the Institutional Animal Care and Use Committee (IACUC) at the University of Alabama at Birmingham (APN: IACUC-21123). This study is reported in accordance with the ARRIVE guidelines (Animal Research: Reporting of In *Vivo* Experiments) to ensure transparent and comprehensive reporting of animal research methods and findings and performed in accordance with the National Institute of Health guidelines for the care and use of laboratory animals.

Male C57BL/6J mice at 6–10 weeks of age (20–25 g) were group housed on a 12 h light dark cycle with *ad libitum* access to food and water. Animals were injected (s.c.) with 10 nmol/kg semaglutide, 10 nmol/kg [d-Ala^2^] GIP_1–42_, 10 nmol/kg semaglutide plus 10 nmol/kg [d-Ala^2^] GIP_1–42_, or vehicle on the morning of the experiment. One hour following incretin agonist or vehicle administration, urethane (7 mg/kg of 20% w/v in saline, Sigma; i.p.) anesthesia was administered. To prevent pain or distress, mice undergoing surgical procedures were maintained under deep anesthesia for the duration of the experiment and were not allowed to regain consciousness. Prior to initiating surgery, depth of anesthesia was confirmed by the absence of response to a toe and tail pinch and the presence of deep and regular breathing. Anesthetic depth was monitored frequently (every ∼ 15–30 min). If any signs of pain or anesthesia withdrawal (shallow and at irregular breathing, toe/tail response, whisker twitching) were observed, additional anesthesia was immediately administered. If distress accelerated or continued after 30 min, the animal was humanely euthanized. At the conclusion of the procedure, euthanasia was performed by cervical dislocation followed by a secondary method or decapitation.

The carbon fiber microelectrode was implanted in the caudal lateral septum (LSc; AP: +0.8, ML: −0.3, DV: −2.5) and the electrical stimulating electrode was implanted in the medial forebrain bundle (MFB; AP: −1.6, ML: −1.0, DV: −4.8). Fast scan cyclic voltammetry (FSCV) was used to detect the oxidation and reduction of DA in the LS. Data were collected and analyzed as previously described. Cocaine (10 mg/kg) was administered i.p.

### Electrode Fabrication

Carbon fiber microelectrodes were fabricated by aspirating a carbon fiber (Goodfellow Corporation, Coraopolis, PA, United States) through a glass capillary (0.4 mm internal diameter, 0.6 mm outer diameter, A-M Systems, Carlsborg, WA, United States), and then pulled to a point using a vertical pipet puller (Kopf Instruments, Tujunga, CA, United States). Exposed fibers were cut to 50 μm and nafion (L-Q-1105, Ion Power, New Castle, DE, USA) was electropolymerized on the surface by applying a constant potential of 1 V for 30 s.^14^

### FSCV Data Collection and Statistical Analysis

Fast scan cyclic voltammetry (FSCV) was used to detect the phasic oxidation and reduction of DA in anesthetized mice. All measurements were collected using a Dagan potentiostat (Dagan Corporation, Minneapolis, MN), custom built hardware interfaced with PCIe 6431 and PCI 6221 DAC/ADC cards (National Instruments, Austin, TX), and a Pine Research headstage (Pine Research Instruments, Durham, NC). WCCV 4.0 software (Knowmad Technologies LLC, Tucson, AZ) was used to apply the DA waveform (−0.4 to 1.3 V to – 0.4 V) at a scan rate of 400 V/s and a frequency of 10 Hz. DA release was evoked using a biphasic electrical stimulation (60 Hz, 360 μA, 2 ms in width) for 2 s through a linear current stimulus isolator (NL800A Neurolog, Medical Systems Corp, Great Neck, NY).

Data was collected and filtered using WCCV software (zero phase, Butterworth, 2 kHz low pass filter). Mice received s.c. injections of either vehicle, semaglutide, [d-ala]_1–42_GIP (blue), or a combined dose of semaglutide and [d-ala]_1–42_GIP (purple) 5 h. prior to cocaine (10 mg/kg in saline, Sigma, i.p.) administration. Three control FSCV measurements were collected and averaged, once every 10 min. Then cocaine was administered, and FSCV measurements were taken at 30 min after cocaine administration. The obtained currents were converted to concentration using a previously reported calibration factor (0.0625 μM/nA).^14^ Maximum amplitude and area under the curve (AUC) were evaluated using Clampfit software (Clampfit 10.6, Molecular Devices, San Jose, CA). Clearance rate (*t*_1/2_) of the decay trace was calculated by fitting an exponential decay curve after the maximum amplitude of the release using Analysis Kid software.^14^

### RNAscope Tissue Preparation and Imaging

Mice were euthanized by CO_2_ and cervical dislocation, and the brains were rapidly removed and immersed in 2-methylbutane and chilled on dry ice for 30 s. Brains were stored in −80 °C until sectioning. Ten μm coronal sections were sliced at −20 °C using a Leica CM1850 cryostat (Deer Park, IL). Brain sections containing the LS (AP: 0.62–1.0) were mounted onto frosted microscope slides and stored at −80 °C until staining.

Following the manufactures recommended protocol, brain sections were stained using the RNAscope Multiplex Fluorescent v2 assay kit (323110, ACD Bio, Newark, CA) and the RNAscope 4-plex ancillary kit (323110, ACD Bio). Channels were matched with genes according to the relative expression level for the following probes (ACD Bio): DAPI, Mm-Drd2-C2 (406501-C3), Mm-Glp1r-C2 (418851-C2), and Mm-Gipr-C3 (319121-C3). For *Drd2* and *Glp1r*, the data presented derive from 2 male and 2 female mice with each mouse quantified from at least LS slices across the rostral to caudal axis. For *Gipr* and *Glp1r*, the data presented derive from three male mice with each mouse quantified from 1 slice. Stained sections were imaged using the BZX800 Keyence Microscope and stitched using the BZ-X800 Keyence Analyzer Software. Images were background subtracted and preprocessed into single and merged channels using Fiji. Background subtracted and preprocessed images were used as inputs to Cellprofiler. DAPI+ cells containing *Glp1r*, *Drd2*, or *Gipr* were segmented and counted using Cellprofiler and quantified in Graphpad Prism.

### Statistical Analyses

All statistical tests were performed using GraphPad Prism (10.3) and statistical significance is expressed as *p* < 0.05. Sample distributions are described as mean ± SEM, unless stated otherwise. Differences in maximum amplitude, clearance rate and AUC between groups were tested for significance using a two-way analysis of variance (ANOVA) and Fisher’s Least Significant Difference posthoc multiple comparisons test. N = 5 mice were used for each of the four FSCV experimental groups (total n = 20 mice): vehicle, semaglutide, [d-Ala^2^]­Gip_1–42_, and combination dose (semaglutide, +[d-Ala^2^]­Gip_1–42_). Each mouse received only one of the 4 treatment groups; however all mice received cocaine. All animals were purchased from Jackson Laboratories and aged matched.

#### *Semaglutide (Eli Lilly)*: * = *p* ≤ 0.0368; ** = *p* ≤ 0.0047

For max amplitude, two-way ANOVA analysis indicated a significant group effect due to vehicle or semaglutide injections (F­(1,8) = 7.275, p = 0.0272) and an effect due to cocaine administration (F­(1,8) = 14.83, p = 0.0049). There was no interaction effect (F­(1,8) = 0.081, p = 0.7823). For *t*_1/2_ values, two-way ANOVA analysis indicated no effect due to vehicle or semaglutide groups (F­(1,8) = 0.212, p = 0.6572) or due to cocaine administration (F­(1,8) = 3.979, p = 0.0812). There was no interaction effect (F­(1,8) = 3.626, p = 0.0933). Upon Fisher’s LSD posthoc test, animals administered vehicle exhibited significant slowing following cocaine administration (p = 0.0248). Two-way ANOVA analysis for evaluating AUC indicated a significant main group effect between vehicle and semaglutide administration (F­(1,8) = 5.775, p = 0.0430), and a group effect of cocaine administration (F­(1,8) = 20.36, p = 0.0020). There was no interaction effect (F­(1,8) = 0.946, p = 0.3590).

#### [d-Ala^2^] GIP_1–42_(Genscript): * = p ≤ 0.049; ** = p ≤ 0.0057; **** = p ≤ 0.0001

For maximum amplitude quantification, two-way ANOVA analysis indicated a significant difference due to vehicle and [d-Ala^2^] GIP_1–42_ administration groups (F­(1,8) = 13.19, p = 0.0067), as well as a group effect due to cocaine administration (F­(1,8) = 78.20, p = 0.0001). However, there was no interaction effect (F­(1,8) = 4.717, p = 0.0616). For reuptake decay values, two-way ANOVA analysis indicated no group effect because of vehicle or [d-Ala^2^] GIP_1–42_ administration (F­(1,8) = 1.970, p= 0.1981). However, there is a main group effect following cocaine administration (F­(1,8) = 9.935, p = 0.0136). There was no interaction effect (F­(1,8) = 0.014, p = 0.9078). Two-way ANOVA analysis for evaluating AUC indicated a significant difference between vehicle and semaglutide groups (F­(1,8) = 7.233, p = 0.0275), as well as a cocaine administration effect (F­(1,8) = 21.54, p = 0.0017). There was no interaction effect (F­(1,8) = 3.130, p = 0.1148).

#### Semaglutide + [d-Ala^2^] Gip_1–42_: * = p ≤ 0.04; ** = p ≤ 0.005; *** = p ≤ 0.0009; **** = p ≤ 0.0001

For quantification of max amplitude, two-way ANOVA analysis indicated a significant main effect vehicle and combined dose administration groups (F­(1,8) = 21.76, p = 0.0016) cocaine administration effect (F­(1,8) = 45.08, p = 0.0002). There was a significant interaction effect (F­(1,8) = 9.410, p = 0.0154). For *t*_1/2_ reuptake values, two-way ANOVA analysis indicated a significant effect due to the administration of vehicle and combined semaglutide-GIP injections (F­(1,8) = 10.92, p = 0.108), as well as an effect due to cocaine administration (F­(1,8) = 8.637, p = 0.0187). There was no interaction effect (F­(1,8) = 2.297, p = 0.1681). Two-way ANOVA analysis evaluating AUC indicated a significant group effect due to vehicle or combined dose administration (F­(1,8) = 10.52, p = 0.0118), as well as a group effect due to cocaine administration (F­(1,8) = 27.76, p = 0.0008). There was no interaction effect (F­(1,8) = 3.979, p = 0.0812).

## Data Availability

The data sets used and/or analyzed during the current study are available from the corresponding authors on reasonable request.
